# Electrospun Multiple-Chamber Nanostructure and Its Potential Self-Healing Applications

**DOI:** 10.3390/polym12102413

**Published:** 2020-10-20

**Authors:** Yubo Liu, Xinkuan Liu, Ping Liu, Xiaohong Chen, Deng-Guang Yu

**Affiliations:** School of Materials Science & Engineering, University of Shanghai for Science & Technology, Shanghai 200093, China; 172442562@st.usst.edu.cn (Y.L.); liup516@163.com (P.L.); cxh992@163.com (X.C.); ydg017@usst.edu.cn (D.-G.Y.)

**Keywords:** multifluid electrospinning, core-sheath nanofiber, complicated nanostructure, epoxy resin, polymeric composite

## Abstract

To address the life span of materials in the process of daily use, new types of structural nanofibers, fabricated by multifluid electrospinning to encapsulate both epoxy resin and amine curing agent, were embedded into an epoxy matrix to provide it with self-healing ability. The nanofibers, which have a polyacrylonitrile sheath holding two separate cores, had an average diameter of 300 ± 140 nm with a uniform size distribution. The prepared fibers had a linear morphology with a clear three-chamber inner structure, as verified by scanning electron microscope and transmission electron microscope images. The two core sections were composed of epoxy and amine curing agents, respectively, as demonstrated under the synergistic characterization of Fourier transform infrared spectroscopy, thermogravimetric analysis (TGA), and differential scanning calorimetry. The TGA results disclosed that the core-shell nanofibers contained 9.06% triethylenetetramine and 20.71% cured epoxy. In the electrochemical corrosion experiment, self-healing coatings exhibited an effective anti-corrosion effect, unlike the composite without nanofibers. This complex nanostructure was proven to be an effective nanoreactor, which is useful to encapsulate reactive fluids. This engineering process by multiple-fluid electrospinning is the first time to prove that this special multiple-chamber structure has great potential in the field of self-healing.

## 1. Introduction

As a cheap adhesive, epoxy resin is widely used in industrial production because of its excellent chemical properties and good sealing performance [1,2,3]. However, the high cross-linking density of cured epoxy resin leads to its brittleness, which affects the lifespan of the material. Self-healing materials, which apply the self-healing mechanism of biological wounds, can extend the lifespan of existing materials and reduce the costs associated with repair and maintenance, thus attracting extensive attention [4,5,6]. A living organism releases a certain substance and then transports it to the damaged part through the blood capillary for self-healing. For example, the skin can recover within a few days after being scratched because of the function of platelets in the human body [7].

Inspired by this interesting natural phenomenon, White and his coworkers reported an epoxy-based microcapsule as a self-healing system in 2001 [8]. The healing agent from cracked microcapsules flowed to the damaged area through capillary action for curing. Later, people found that composite materials containing a bionic microvascular network can simultaneously repair multiple cracks [9,10]. However, both microcapsules [11,12,13,14] and microvascular network [15,16,17] have a tedious preparation process, thereby greatly limiting their potential applications. Thus, how to simplify the preparation process and endow self-healing materials with good self-healing performance has drawn increasing attention [18,19].

The concept of electrospun fiber-based self-healing materials was proposed and demonstrated firstly by Braun et al. in 2010 [20]. Electrospinning can encapsulate the reaction healing agent into nanofibers with little negative influences on the mechanical properties of materials in a single-step and a straightforward manner. Braun et al. confirmed that delivering the encapsulated reactive liquid agent into the gap area is a feasible way to prevent the spread of cracks and restore the mechanical properties of the damaged material. Nanofibers that contain a healing agent can be embedded into the epoxy matrix to provide it with a good self-healing ability. These nanofibers can also act as the reinforcing elements to improve the matrix materials’ mechanical performances such as tensile strength and high modulus [21,22]. When cracks occur, a healing agent and a curing agent from the broken fibers will be released simultaneously to contact with each other and react to cure the crack. Doan et al. demonstrated that the use of polyvinyl alcohol (PVA) to encapsulate two parts of dimethylsiloxane (DMS) was feasible [23]. Lee et al. reported that a blend solution of Poly(vinylidene fluoride) (PVDF) and polyethylene oxide (PEO) had a good effect on encapsulating the reactive DMS and its healing agents [24,25]. Unfortunately, much room for improvement remains for traditional working processes, such as layered electrospinning and double coaxial electrospinning [26], in terms of facile preparation, accurate loading of the healing and curing agents, and utilization efficiency. Most recently, Yildiz et al. used three-fluid electrospinning to prepare self-healing materials. A new sort of tri-layer nanofibers being loaded with two reactive healing agents at the same time was reported to be able to maximize the efficiency of healing agents [27].

During the past decade, the traditional single-fluid electrospinning has been quickly shifting to multifluid working processes for creating nanofibers and nanocomposites with complex structural characteristics [28,29,30,31]. For successful multifluid electrospinning, reasonable selections of working parameters such as flow rates, voltage, and collection distance are important, as in the traditional single-fluid blending electrospinning. However, this process involves new parameters that increase the difficulty of implementation [32,33,34]. Among all the kinds of electrospun complex nanostructures, the core-shell and Janus structures are the most fundamental ones. Other, more complex fiber structures that have emerged can be regarded as a combination and/or a derivative of these two basic prototypes [35].

Inspired by natural phenomena, such as the eyes of panda, vascular bundles of corn stalks, blood capillaries, and nerve endings, a brand-new structural spinneret was designed by Yu et al. [36]. On the basis of the structural spinneret, a new three-fluid electrospinning process can be further developed, by which the similar nanostructures can be generated with the use of the spinneret’s nozzle as a template [37]. This novel multifluid electrospinning method, which can be called real one-step synthesis, greatly simplifies the preparation process compared with the triaxial electrospinning mentioned above. The parallel distribution of the two core sections in a separate manner within the nanoscale by the shell layer is expected to achieve its potential self-healing effect through an accurate and simultaneous release of a healing agent and a curing ingredient.

On the basis of the abovementioned knowledge, epoxy resin and its amine-based curing agent were simultaneously encapsulated into the electrospun structural nanofibers as two separate cores by a new multifluid electrospinning method for the first time in this work. It is a real, one-step process, and the simultaneous release of two kinds of reactive healing agents gives full play to the advantages of multi-chamber nanostructures. The raw materials can be fully utilized to play a role in environmental protection and biomedical applications [38,39,40,41]. The flexibility of polyacrylonitrile (PAN) increases the possibility of successfully encapsulating the healing agent, and amine curing agents allow epoxy resin to be cured quickly at room temperature. The prepared fibers were subjected to a series of characterizations, and the self-healing performances of the fiber in a bulk epoxy resin were verified by artificial scratches.

## 2. Materials and Methods

### 2.1. Materials

PAN (*M*_w_ = 50,000 g/mol) was purchased from Shanghai Chemical Fibers Institute (Shanghai, China). Epikote-828 (*M*_w_ = 392 g/mol, epoxide number 0.51) used in this study was supplied by Hexion (Columbus, Ohio, USA), and the amine-based curing agent triethylenetetramine (TETA) was supplied by China National Medicines Corporation Ltd. (Shanghai, China). The matrix was a mixture of epoxy resin and amine curing agent at a ratio of 100:12 without further modification. The spinning solvent *N,N*-dimethylacetamide (DMAC) and acetone was purchased from Sinopharm Chemical Reagent Co., Ltd. (SCRC; Shanghai, China).

### 2.2. Electrospinning

The 10% *w/v* PAN was dissolved in a mixture of DMAC and acetone (3:1, v:v), which was stirred at a constant magnetic force for 24 h at 60 °C and then used as an electrospun nanofiber shell. The 75% *w/v* epoxy resin and amine curing agent, which were separately dissolved in a mixture of the above two solvents, were prepared as core layers, which were separately supplied to the core needle. The three solutions were controlled by separate pumps (KDS100, Cole-Parmer^®^, Vernon Hills, IL, USA). In the electrospinning process, fibers were collected on aluminum foil placed 15 cm away from the spinning nozzle, and the operating voltage was 15 kV, powered by a power supply (ZGF60 kV/2 mA, Wuhan Hua-Tian Co., Wuhan, China). The homemade spinneret consisted of a sheath steel tube (2.02 mm for inner diameter) and two metal capillaries (0.26 mm for inner diameter).

### 2.3. Characterization

#### 2.3.1. Morphology

The surface morphology of prepared fibers was observed using a field emission scanning electron microscope (FESEM; FEI Quanta 450 FEG, Hillsboro, OR, USA). Prior to a microscope observation, the samples were sputter-coated by platinum under a vacuum-nitrogen atmosphere to render them electrically conductive. The average diameter was calculated by selecting at least 100 fibers in FESEM images using NIH Image J 1.52a software (National Institutes of Health, Bethesda, MD, USA). The internal structure of the fiber was observed by transmission electron microscope (TEM; Hitachi, Japan).

#### 2.3.2. Chemical and Thermodynamic Analyses of the Nanofibers

Fourier transform infrared (FTIR) spectroscopy of samples in the range of 500–4000 cm^−1^ was conducted by using a Bruker Optics VERTEX 80/80v FTIR spectrometer (Ettlingen, Germany) with a resolution of 0.06 cm^−1^. The appearance of related characteristic functional groups observed by FTIR proved that the healing agent was successfully encapsulated into the nanofibers.

#### 2.3.3. Thermal Analysis

The amount of encapsulation epoxy resin and amine curing agent in the core–shell nanofibers were evaluated by conducting a thermogravimetric (TGA) experiment with the use of a Pyris 1 TGA analyzer from PerkinElmer (Boston, MA, USA). Prior to this step, methanol was used to pretreat the fiber to clean up the healing agent that was not successfully encapsulated, to reduce interference with the experimental results. The temperature range of the TGA experiment was from room temperature to 600 °C with a heating rate of 10 °C min^−1^, and the nitrogen gas flow rate was 20 mL min^−1^. The curing behavior of epoxy and the amine curing agents encapsulated in the core-shell nanofibers were observed by using a differential scanning calorimetry (DSC, NETZSCH STA 449 F3 Jupiter, Selb, Germany). Prior to the experiment, three vacuum and purge gas cycles were carried out. The constant heating rate was set at 30 °C min^−1^ from 30 °C to 350 °C under argon gas with a flow rate of 20 mL min^−1^.

#### 2.3.4. Electrochemical Corrosion

Electrochemical corrosion was controlled by computer-assisted, three-electrode electrochemical cells. In this system, Pt is used as the auxiliary electrode, the reference electrode is Ag/AgCl3, and the steel sheet containing the self-healing coating or the comparative sample is used as the working electrode. The samples were tested using linear sweep voltammetry in an aqueous solution of 3.5% *w/v* NaCl, with a constant speed of 20 mV/s in a linear scan in the range of −0.7–0.7 V.

## 3. Results and Discussion

### 3.1. Electrospinning

Unlike coaxial electrospinning, the electrospinning used in this study uses an additional metal capillary in the metal tube, and the two metal capillaries are distributed in parallel in the internal structure. This property plays an encouraging role in the field of self-healing materials. However, the complex multiple-fluid process is not easy to realize because many parameters need to be set. Considering the unspinnability of epoxy and TETA and the opposite surface tension, the inappropriate parameters can lead to poor results, such as spatter and intermittent spinning caused by the excessive relative flow rate of core liquid. The desired fiber can be prepared only after the parameters reach a dynamic balance under the ingenious control of parameters, such as the different solvent evaporation rates of working fluids, the concentration of the solution, the flow rate, and the relationship between the mixed droplet and the applied voltage. To ensure that PAN perfectly encapsulates the two components of the healing agent, the appropriate parameter settings [42] were applied to produce electrospun nanofiber with a uniform diameter, certain directionality, and smooth surface morphologies. Yu’s research on the relationship between Taylor cone, a straight fluid jet, and unstable regions provides a clear process–property relationship to study the interaction between energy and fluid [43,44]. In this study, two fibers—one monolithic and one triaxial—were prepared. More detailed experimental parameters are presented in Table 1.

**Figure 1:**
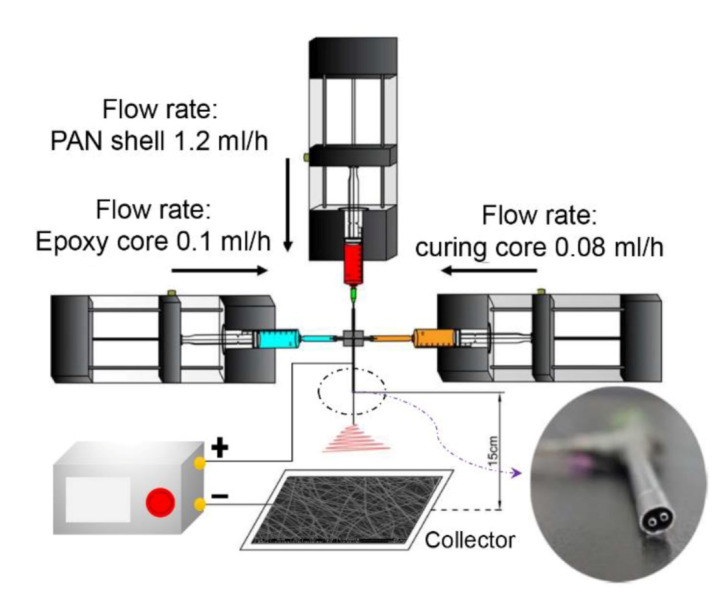
Schematic illustration of the electrospinning polyacrylonitrile (PAN) multiple-chamber nanostructure nanofibers process. The inset shows the internal structure of the homemade spinneret.

As shown in Figure 1, electrospinning was carried out under the synergistic effect of three pumps that controlled different spinning solutions. Under the appropriate applied voltage, the droplets at the nozzle formed a jet stream and were gradually stretched into nanometer-sized fibers under bending and whipping, which were then randomly deposited on aluminum foil. As illustrated in Figure 1, the epoxy resin monomer and its amine curing agent were encapsulated as core layers, respectively. A homemade spinneret is shown in Figure 1. The spinnerets, which are utilized to create electrospun nanostructures in the forms of linear fibers, can also be exploited to prepare structures in the forms of particles by using electrospraying in the future [45,46,47,48].

**Figure 2:**
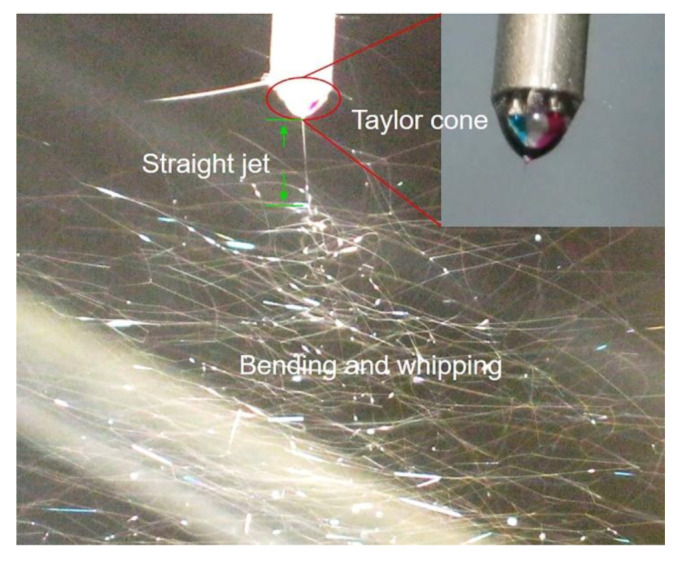
Digital image of the advanced, multi-fluid electrospinning process, including Taylor cone, jet, and bending. The inset picture shows the visualization process by dyeing the two healing fluids.

Figure 2 shows the digital photograph of the electrospinning process. The cores were color-marked to visualize the fiber-forming process. The dye gives the epoxy resin a blue color and a red color for TETA. The complicated Taylor cone (with two inner fluids encapsulated by the outer solvent) is depicted in Figure 2, followed by the direct jet zone and the whipping bending. The detail of the complex Taylor cone in the illustration indirectly proves the applicability to wrap two kinds of healing agents. At the same time, no contact reaction exists between the two reactive healing agents in the advanced multi-fluid electrospinning process.

### 3.2. Morphological Characterization

**Figure 3:**
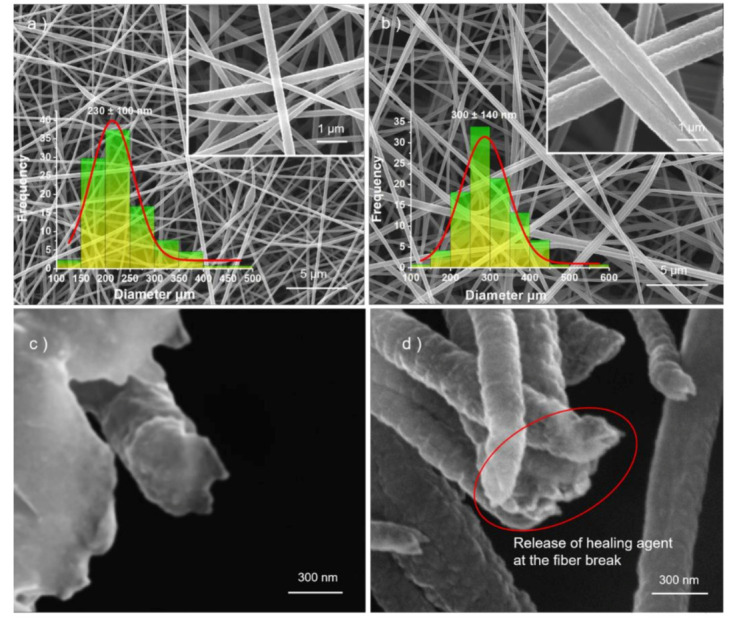
SEM images of fiber distinguished by different size: (**a**) PAN fibers; (**b**) the special core-shell fibers; (**c**) the broken PAN fibers; (**d**) the reactive healing agent solidifies at the broken core-shell fibers.

A common feature of the two fibers shown in Figure 3 is that the fibers are uniform in diameter and have no bead or spindle structure. First, the monolithic fibers, F1, were prepared by single-fluid electrospinning. F2, shown in Figure 3, is core-sheathed nanofibers with special structures. The average diameters, related to the two unspinnable working fluids encapsulated by PAN and the fluid rate, are shown in Table 1. Comparing with other PAN core-shell nanofibers in the literature [49], the nanofibers prepared by the multiple-fluid electrospinning process had a smaller diameter of 300 ± 140 nm, which is larger than the average diameter of PAN (230 ± 100 nm). The adhesion phenomenon between fibers can be attributed to the viscosity of the epoxy resin, which was not successfully encapsulated. Comparing with the smooth fracture surface in Figure 3c, the adhesive lumps in Figure 3d give a hint about the successful release of the healing agents from the separate cores of the nanostructures and the late curing reactions. The red circle exhibits the cured epoxy resin after the release of the healing agent.

**Figure 4:**
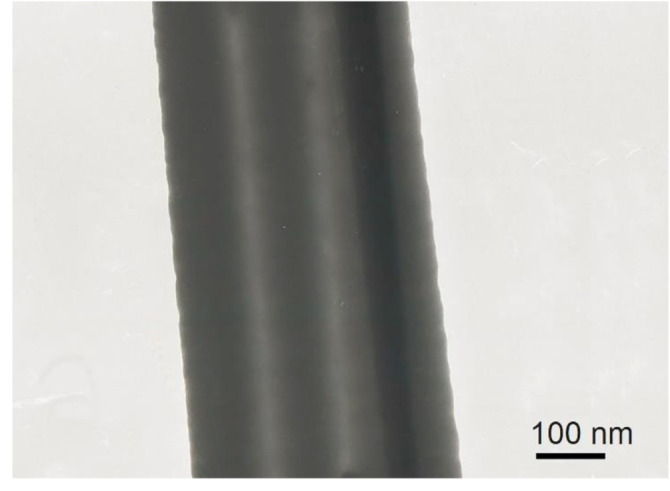
TEM image of PAN-epoxy-curing agent core-shell nanofibers.

The structure of the core–shell nanofibers were further observed through TEM images. The fibers were deposited on paper, which had a gap of a certain width, for a few seconds and then transferred to a copper mesh to prepare the TEM sample. As expected, a clear interlayer structure with dark inner layers and a light gray outer layer was obtained, as shown in Figure 4. Different shadows in the inner layer were represented by two healing agents. This condition is undoubtedly the most intuitive evidence that the two reactive healing agents were successfully encapsulated.

### 3.3. Chemical Structure of Core-Shell Nanofibers

**Figure 5:**
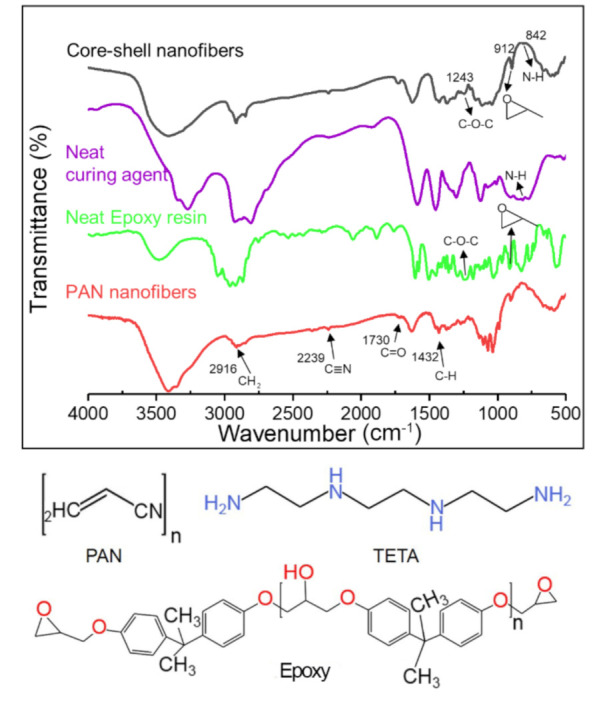
Fourier transform infrared (FTIR) spectra of PAN fibers, neat epoxy resin, neat curing agent, and core-shell nanofibers.

Figure 5 shows the IR spectrum of the PAN nanofiber, and epoxy, TETA, and core-shell nanofibers are among them. First, the IR of the PAN nanofiber shows four typical peaks at 2916 cm^−1^, 2239 cm^−1^, 1730 cm^−1^, and 1432 cm^−1^, which were attributed to the –CH_2_ absorption, N stretching bands, C=O stretching bands, and CH bands, respectively. For the neat epoxy, two typical absorption bands were clearly observed at 1243 and 912 cm^−1^, representing the characteristic functional groups of C–O–C and epoxide, respectively. For neat TETA, peaks at 842, 1588, and 3340 cm^−1^ were attributed to the N–H group. The structure of PAN, TETA, and epoxy are shown at the end of Figure 5. Considering the coincide of the characteristic peaks of PAN and epoxy resin, and the few amounts of epoxy and triethylenetetramine, the characteristic peaks of the two healing agents in the core-shell fibers IR was not obvious. PAN, as a good container [50,51,52] in encapsulating the two reactive healing agents, will be further judged by thermal stability.

### 3.4. Thermal Analysis

**Figure 6:**
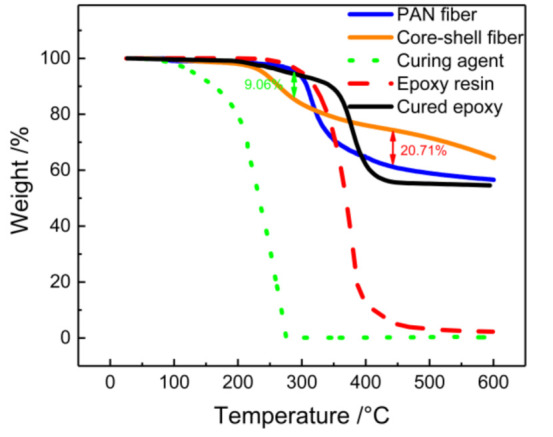
Typical thermogravimetric (TGA) curves for core-shell fiber, PAN fiber, neat epoxy, neat TETA, and cured epoxy.

The thermal stability of core–shell nanofibers is an important index to determine the ability to repair resin and the limitation of the application range. As a technique for measuring the relationship between mass loss and temperature, TGA can infer the fiber composition and the dose of reactive healing agent for nanofiber packaging according to the change of the curve. Figure 6 shows a typical curve of core–shell nanofibers, PAN nanofibers, neat epoxy, neat TETA, and cured epoxy. Two stages of mass loss occurred in the case of PAN fiber. The first rapid decline step began at 300 °C and ended at 350 °C, which was attributed to the volatile products produced by PAN pyrolysis, such as NH_3_, CH_3_CN, and HCN. The second stage of PAN decomposition occurred between 350 and 500 °C. For epoxy resin and cured epoxy, the decomposition temperature range was 280–475 °C, and the loss of mass was mainly due to the decomposition of phenolic compounds in epoxy resin. TETA had only one step during thermal decomposition from 115 °C to 276 °C. Compared with the PAN fibers, core–shell nanofibers showed less mass loss, as presented in Figure 6. This can be attributed to the decomposition by PAN from 300 to 350 °C, which led to a small amount of contact curing of TETA and EP. The decline from 200 to 276 °C was attributed to the TETA from core-shell nanofibers, which means the core-shell nanofibers contained 9.06% TETA. Compared with the rapid decline of epoxy resin in 280–475 °C, the core-shell fibers exhibited a slight state. With the pyrolysis of PAN, a few TETA and epoxy solidified rapidly at high temperature. Considering the stable state of cured epoxy at 430 °C, the difference between core-shell fibers and PAN fibers was attributed to the 20.71% cured epoxy in fibers. This fact proves that the two reactive healing agents were successfully encapsulated into the nanofibers and there was no contact reaction between the two because of the isolation of PAN. According to the electrospinning flow rates, the theoretical contents of epoxy and curing agent in the fibers are 29.41% and 23.53%. The discrepancy between theoretical and actual value suggests that unencapsulated healing agents were removed during washing with methanol.

**Figure 7:**
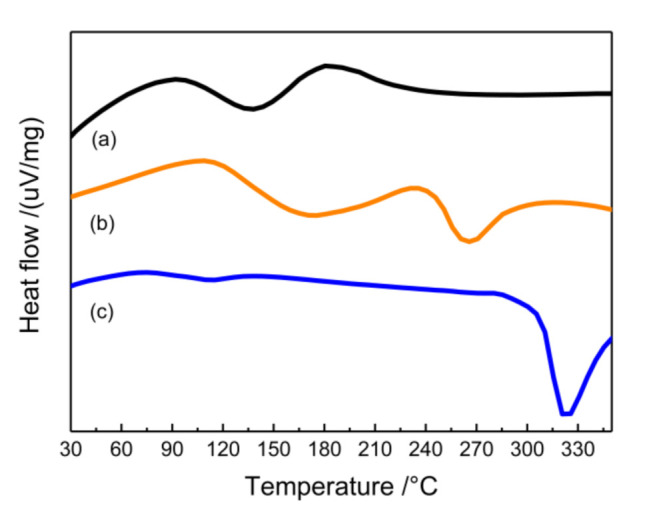
Differential scanning calorimetry (DSC) curves for core-shell fiber and PAN fiber. (a) The reaction between epoxy and the amine healing agent; (b) the multiple-chamber, core-shell nanofibers; (c) the PAN nanofibers.

The cross-linking reaction between epoxy and the amine healing agent is essentially exothermic, and this exothermic process can be well observed by DSC. Figure 7 shows the DSC curves of core-shell fibers and PAN fibers. There was only one obvious exothermic peak of PAN fibers between 325 °C, which was attributed to the cyclization reaction of PAN. For core-shell fibers, the exothermic peak of PAN in the curve of core-shell fibers shifted a lot and occurred at 265 °C, which was attributed to small amounts of epoxy and TETA loaded in PAN. Comparing with the exothermic peak in epoxy resin curing reaction at 138 °C, the curve of core-shell fibers appeared as a similar weak curing peak (a small amount of reactive healing agents in fibers) at 175 °C. This fact shows that the reactive fluid encapsulated in the fiber can be cured, and core-shell fibers have the ability to self-heal, which can be confirmed by the embedded epoxy coating in the next part.

### 3.5. Self-Healing Mechanism

**Figure 8:**
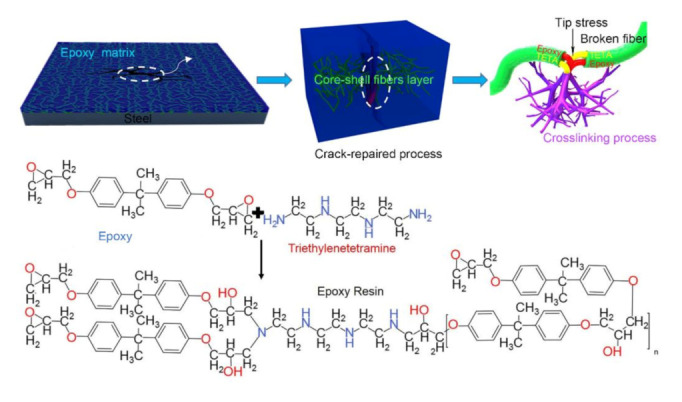
Self-healing schematic of cracks in the multiple-chamber, core-shell nanofiber. The red fiber encapsulates the healing agent, while the green fiber encapsulates the curing agent.

A protective layer can form on the surface of the steel substrate by coating because of the good adhesion of epoxy resin [53,54,55] and its nonreactive nature with metal. Water in the air is blocked by the coating to prevent the corrosion. In this experiment, randomly oriented core–shell nanofibers were collected on steel sheets, and the anti-corrosive coating was prepared by spin coating epoxy resin based on the steel sheet containing fibers. The crack-repaired process is shown in Figure 8. The core–shell fibers were randomly distributed in the epoxy matrix, and fibers near the crack released the healing agent and cured the interlaminar crack. The PAN sheath exhibited a shielding effect, thus providing a protective layer and a physical barrier for epoxy resin and TETA. When cracks occurred in the self-healing coating samples, the tip stress of the crack led to the fracture of the fiber. Then the reactive healing agent inside the fiber flowed to the damage and solidified the crack under capillary action. The chemical mechanism of cross-linking between epoxy and TETA is also shown in Figure 8. Epoxy has good chemical stability and does not cause fiber dissolution and swelling. The purpose of spin coating is to remove excess epoxy resin and form an anti-corrosive protective film with uniform thickness. The ingenious combination of core–sheath fibers and epoxy composites [56] produces a good self-healing ability.

**Figure 9:**
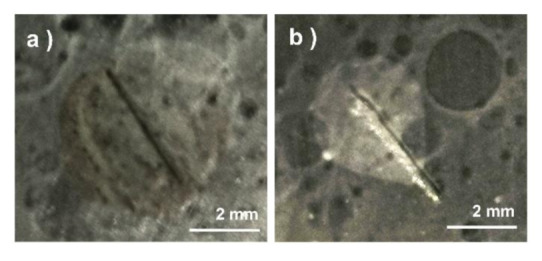
The digital pictures of the artificial scratch coating after corrosion resistance test: (**a**) Pure epoxy coating, (**b**) self-healing coating.

The self-healing behavior of coating can be visualized by electrochemical measurements. Artificial scratches, the depth of which must reach the surface of the steel substrate, needed to be made on the self-healing coating and the control sample by using sharp cutting tools before electrochemical corrosion (Figure 9). The samples should be stored at room temperature for one day to give it enough time to heal. Compared with the obvious corrosion phenomena showed in pure epoxy coating (Figure 9a), the self-healing coating in Figure 9b shows a good anti-corrosion phenomenon. More details about corrosion resistance test will be discussed in the next section.

### 3.6. Corrosion Resistance of Self-Healing Materials

**Figure 10:**
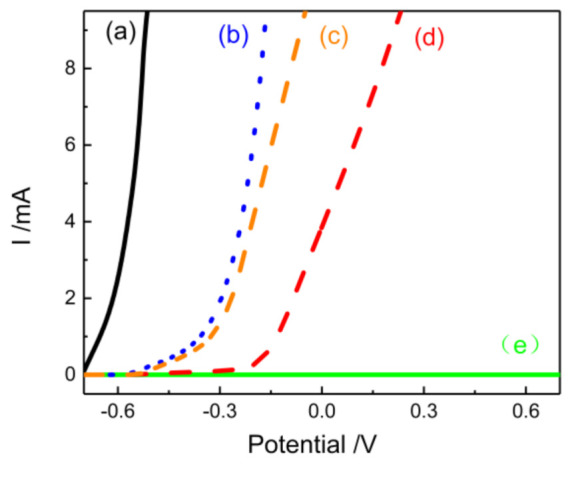
The electrochemical curve of steel substrate (a), epoxy coatings (b), epoxy coatings containing 30-min PAN fibers (c), self-healing composite coatings with different fiber deposition time on the steel substrate: 15-min core-shell fibers (d) and 30-min core-shell fibers (e). When the fiber deposition time was more than half an hour, the current tended to 0 mA and curves coincided, which indicated that self-healing coating are effective.

In Figure 10, the electrochemical corrosion shows that the epoxy composite coating containing enough core–shell fibers had ideal healing characteristics. However, the coating without core–shell fibers did not have the ability to protect after scratches, which corresponded to the significant current in electrochemical measurements (Figure 10). This finding indicates that the healing phenomenon did not occur. Figure 10 shows that fibers in epoxy coatings exhibit a limited current hindrance, while the core-shell fibers have a stronger effect than PAN fibers. The coating that contains core-shell fibers deposited for 15 min showed a certain inhibition of current compared with pure epoxy coating. The significant electrochemical differences of the control samples indicate the feasibility of electrospinning for preparing self-healing materials and the effectiveness of self-healing coatings. This finding also indicates that the core–shell fiber content in the coating was not enough to play a curing role. The fiber layer with a deposition time of more than half an hour in the coating had good self-healing ability, as indicated by the low current passing through the coating. The successful self-healing coating provided the steel substrate to have the ability of secondary anti-corrosion. The first artificial scratches damaged the first line of defense of the coatings. However, its healing ability successfully prevented the current from passing through.

## 4. Conclusions

To prevent crack propagation and increase the service life of materials, this work successfully prepared a novel, multiple-chamber, nano-structural fiber with an excellent healing effect by using multiple-fluid electrospinning, and the self-healing ability of this fiber was verified by scratch coating. The average diameter of the fiber was 300 ± 140 nm and showed a linear morphology characterized by nonstring beads. The SEM diagram successfully observed newly cured epoxy resin after the release of the healing agent at the fiber fracture. The good hierarchical structure of the fiber, which observed by TEM, exhibited that the multiple-chamber nanostructure, as an effective nanoreactor, is a useful way to encapsulate reactive healing agents. The chemical structure and thermodynamic analyses indicated that PAN, as a sheath material, has excellent potential in encapsulating two reactive healing agents at the same time. However, the pyrolysis of PAN caused the reactive fluid to solidify at high temperature. Only 9.06%TETA and 20.71% cured epoxy was obtained from the typical TGA curve. The exothermic peak of the reaction of epoxy on the DSC curve of core-shell fibers suggested the ability of curing in fibers. The complete self-healing ability of core-shell fibers was shown by the electrochemical test of coating with scratches, which can provide good protection for steel substrate. The coating containing fibers that underwent electrospinning for 30 min, which provided secondary corrosion protection to the steel substrate, showed an excellent protective effect. This self-healing behavior is the first time that multi-fluid electrospinning engineering products showed their potential self-healing ability, which fabricated the functional multiple-chamber nanofibers. The ingenious design of structure brings functional products. This study provides a new strategy for the research and development of self-healing materials based on the unique structure of nanofiber formed by advanced electrospinning.

## Figures and Tables

**Table 1:** Key parameters for the electrospinning processes and their products.

No.	Process	F_CE_^a^	F_CT_^b^	F_S_^c^	Morphology ^d^	Diameter
		(mL/h)	(mL/h)	(mL/h)		(nm)
F1	Single	--	--	1	Linear	230 ± 100
F2	Tri-axial	0.1	0.08	1.2	Linear	300 ± 140

^a^ The flow rate of the core epoxy (75 *w/v*%); ^b^ the flow rate of the core triethylenetetramine (TETA) (75 *w/v*%); ^c^ the flow rate of the sheath polyacrylonitrile (PAN) (10% *w/v*).^d^ Linear morphology indicates that fibers were straight and had a certain orientation with few spindles or beads.
